# Sevelamer inhibits the formation of cholesterol gallstones by modulating bile acid metabolism

**DOI:** 10.3389/fphar.2026.1737631

**Published:** 2026-02-10

**Authors:** Shuang Shen, Min Ning, Muhan Li, Shengnan Qian, Xin Ye, Qian Zhuang, Shan Wu, Xinjian Wan, Zhixia Dong

**Affiliations:** 1 Digestive Endoscopic Center, Shanghai Sixth People’s Hospital Affiliated to Shanghai Jiao Tong University School of Medicine, Shanghai, China; 2 Central Lab, Shanghai Sixth People’s Hospital Affiliated to Shanghai Jiao Tong University School of Medicine, Shanghai, China

**Keywords:** bile acid, FXR, gallstone, microbiota, Sevelamer

## Abstract

**Background:**

The purpose of this study was to investigate the effect and mechanism of Sevelamer hydrochloride (Sev) on cholesterol gallstone formation via the intestinal Fxr-Fgf15 signaling pathway in a mouse model.

**Methods:**

A cholesterol gallstone mouse model was established. Mice were divided into groups treated with Sev, Fxr agonist, or controls. The incidence and severity of gallstones, along with liver/body weight ratio, were recorded. Total cholesterol (TC) and total bile acid (TBA) levels were measured. Biliary cholesterol supersaturation index (CSI) was calculated. Serum ALT and AST levels were quantified by ELISA. The expression of Fxr-Fgf15 pathway-related molecules and bile acid transporters were detected by RT-PCR and Western blot. Targeted bile acid metabolomics characterized ileal bile acid profiles, while metagenomics assessed gut microbiota alteration.

**Results:**

Sev treatment reduced hepatic lipid deposition, lowered biliary CSI, attenuated gallbladder wall thickening, improved liver function, and decreased TC levels. Mechanistically, Sev inhibited the intestinal Fxr-Fgf15 pathway, promoting hepatic bile acid synthesis and altering ileal bile acid composition. Fxr agonist reversed these effects, increasing Fgf15/Shp expression, suppressing bile acid synthesis, elevating CSI, and partially restoring gallstone susceptibility. Sev reshaped gut microbiota diversity, reducing Blautia and enriching Bacteroidales and Roseburia at genus level. Concurrently, Sev modulated the ileal bile acid pool, decreasing Fxr-activating bile acids and increasing Fxr-antagonizing bile acids. Microbiota-bile acid correlation analysis highlighted significant associations between specific taxa and bile acid profiles.

**Conclusion:**

Sev might prevent cholesterol gallstone formation by inhibiting the intestinal Fxr-Fgf15 pathway, promoting hepatic bile acid synthesis, reducing biliary cholesterol supersaturation, and restoring gut microbiota balance.

## Graphical Abstract

**Figure:**
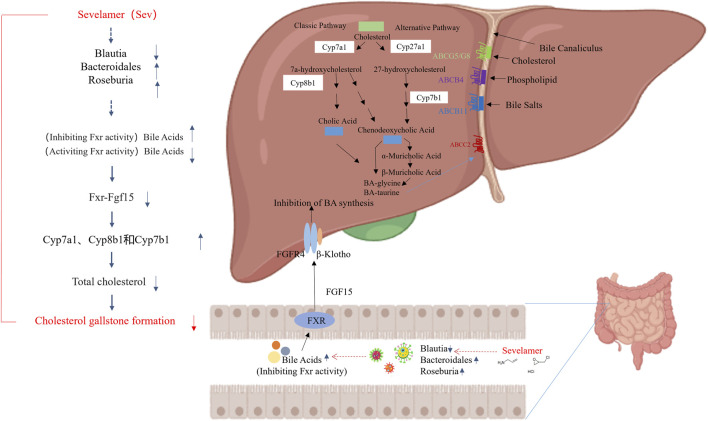

## 1 Introduction

Cholesterol gallstones (CGs), a prevalent global health concern affecting 10%–15% of the worldwide population (Diehl, 1991; Lammert et al., 2016), are associated with potentially life-threatening complications including acute cholecystitis, cholangitis, biliary pancreatitis, and biliary malignancies (Gurusamy and Davidson, 2014; Lammert et al., 2016; Ruhl and Everhart, 2011). Despite significant clinical implications, current therapeutic strategies—surgical intervention and pharmacological management—remain suboptimal, highlighting the urgent need for developing safer, more effective, and cost-efficient preventive and therapeutic agents.

The pathogenesis of CGs involves a complex interplay of genetic predisposition and environmental factors, particularly metabolic dysregulation and gut microbiota alterations. Central to this process is cholesterol supersaturation resulting from disrupted homeostasis between bile acids (BAs), phospholipids, and cholesterol. Under physiological conditions, these components maintain cholesterol solubility through micelle formation. Imbalances in this triad promote cholesterol crystallization and subsequent gallstone formation (Di Ciaula et al., 2018; Wang et al., 2009), though precise molecular mechanisms remain incompletely understood (Hu et al., 2022).

Emerging evidence implicates the farnesoid X receptor (Fxr), a nuclear receptor family member serving as the primary BA sensor, in cholesterol metabolism regulation. Expressed in hepatic, intestinal, and adipose tissues (Makishima et al., 1999; Parks et al., 1999; Wang et al., 1999), Fxr activation through its natural BA ligands initiates a negative feedback loop. When Fxr is activated, hepatic small heterodimer partner (Shp) and intestinal fibroblast growth factor 15 (Fgf15) are induced, which further inhibits the expression of BA synthesis enzymes. This regulatory mechanism suppresses BA synthesis enzymes (Cyp7a1 and Cyp8b1) (Degirolamo et al., 2014), thereby maintaining BA homeostasis.

Sevelamer hydrochloride (Sev), a non-absorbable hydrophilic polymer clinically employed as a phosphate binder for chronic kidney disease patients (Goldsmith et al., 2008), demonstrates intriguing metabolic effects beyond its primary indication. Pharmacokinetic studies confirm its complete fecal excretion without systemic absorption, with clinical trials establishing an excellent safety profile independent of dosage (Brezina et al., 2004; Rosenbaum et al., 1997).

Notably, emerging preclinical evidence suggests Sev modulates BA metabolism and lipid profiles, reducing low-density lipoprotein cholesterol and attenuating hepatic steatosis in lithogenic diet (LD)-fed murine models (Braunlin et al., 2002; Burke et al., 2001; Tsuji et al., 2020). Histopathological evaluations further demonstrate that Sev administration significantly alleviates hepatic steatosis, inflammation, and fibrotic deposition (Chen et al., 2025). The favorable BAs binding properties of Sev offer a compelling mechanism for its ability to reduce cholesterol levels in both hemodialysis patients and healthy volunteers (Braunlin et al., 2002). In patients with gallstones, the diversity of intestinal microorganisms decreases, and beneficial bacteria are reduced (Wu et al., 2013). Transplanting the intestinal microbiota of patients with gallstones into mice can promote the occurrence of gallstones in the mice (Hu et al., 2022), activate intestinal Fxr, and inhibit the expression of liver Cyp7a1 (Hu et al., 2022). Sev can inhibit intestinal Fxr (McGettigan et al., 2016; Tsuji et al., 2020), and simultaneously increase the α-diversity of the intestinal flora in mice (Tsuji et al., 2020). It indicates that Sev has a regulatory effect on the intestinal flora. Recent findings indicate intestinal Fxr pathway inhibition by Sev (McGettigan et al., 2016; Tsuji et al., 2020), though its anti-lithogenic mechanisms remain poorly characterized. We assume that Sev may regulate the BAs profile by modulating the intestinal microbiota, and further inhibits intestinal Fxr, thereby promoting the conversion of cholesterol in the liver to BAs, reducing liver cholesterol levels and thereby preventing the occurrence of CGs.

This study investigates Sev’s potential to mitigate LD-induced CG formation in mice, with particular emphasis on elucidating its molecular mechanisms through comprehensive analysis of cholesterol-BA axis regulation and Fxr signaling modulation.

## 2 Materials and methods

### 2.1 Animal ethics

All animal experiments were conducted with the “3R” concept (reduction, replacement, and refinement) and a protocol approved by the Animal Care and Use Committee of Shanghai Sixth People’s Hospital Affiliated to the Shanghai Jiao Tong University School of Medicine (No.2025-0664).

### 2.2 Feeding experiment

C57BL/6J mice (male, aged 6 weeks, approximately 20 g, GemPharmatech, China) were fed with chow diet (LAD0011, Nantong, Jiangsu, China) to adapt to the environment for 1 week, and then mice were randomly separated into two groups and fed respectively with chow diet and LD (TP 28900 containing 15% fat, 1.25% cholesterol, and 0.5% CA, Nantong, Jiangsu, China) for 6 weeks. All mice were maintained in a specific pathogen-free (SPF) facility under controlled environmental conditions: temperature of 22 °C ± 1 °C, humidity of 55%–65%, and a 12-h light-dark cycle.

### 2.3 Animal treatment

The bedding was replaced before the sample collection, and the mice were fasted for 12 h. The eyeballs of mice were removed under anesthesia by intraperitoneal injection of pentobarbital (1.5%, 10 mL/kg) to get the blood. Then the mice were killed by cervical dislocation. Bile, liver, gallbladder, ileum tissues and gallstones were harvested. Gallbladders and gallstones from each group were photographed and analyzed in the following standards. Gallbladder gallstones of each mouse were specifically evaluated through direct naked-eye observation. The degree of CGs among different groups was graded according to Table 1.

**TABLE 1:** Diet-induced cholesterol gallstone scoring table in mice.

Scoring	Macroscopic appearance of gallbladder
0	Bile clarification in gallbladder
1	The bile in the gallbladder is slightly cloudy, and a small amount of cholesterol crystals can be seen
2	The bile in the gallbladder is cloudy, and more than 10 cholesterol crystals can be seen
3	The bile in the gallbladder is cloudy, and cholesterol crystals occupy no more than half of the gallbladder
4	The bile in the gallbladder is cloudy, and cholesterol crystals are more than half of the gallbladder
5	The bile in the gallbladder is cloudy, and oval or irregular block stones can be seen

### 2.4 Cholesterol analysis

Total cholesterol levels were measured according to the manufacturer’s instructions (Jiancheng Bioengineering Institute, Nanjing, China). Serum and bile can be directly tested for absorbance values. The liver, ileum and other tissues need to be ground to take the supernatant to detect the absorbance value at a wavelength of 562 nm. Total Cholesterol Content was calculated using the following formula: Cholesterol content (mmol/L) = (sample A-blank A)/(standard A-blank A) *C standard (concentration of standard, 5.17 mmol/L). And then multiply by the dilution factor.

### 2.5 BA analysis

Total BA levels were measured according to the manufacturer’s instructions (Jiancheng Bioengineering Institute, Nanjing, China). The absorbance value of bile was detected at a wavelength of 405 nm. Total BA Content was calculated using the following formula: Total bile acid content (µmol/L) = ΔA determination/ΔA standard * concentration of standard (µmol/L), and finally multiplied by the dilution factor.

### 2.6 Phospholipid analysis

Phospholipid levels were detected according to the manufacturer’s instructions (Wako Pure Chemicals, Osaka, Japan). The absorbance of bile was measured at a wavelength of 600 nm. The Cholesterol Supersaturation Index (CSI) was derived by dividing the measured molar percentage of biliary cholesterol by its maximum soluble concentration, with reference values obtained from Carey’s tables at the relevant bile salt molarity.

### 2.7 Haematoxylin and eosin (H&E) staining

Liver samples were collected and fixed in tissue fixative for more than 24 h, processed with ethanol and xylene, and then embedded in paraffin. Samples were sectioned into 4-μm thick (4 μm) sections, de-paraffinised, and stained with H&E for histological examination, and finally observed and acquired images under a microscope.

### 2.8 RNA extraction

The collected tissues (liver, ileum) were placed in a 1.5 mL EP tube and 1 mL of Trizol (R401-01, Vazyme, Nanjing, China) was added. Add 2 steel balls to each EP tube, place the EP tube into a low-temperature grinder, set the parameters to 120 Hz for 60 s, and grind and crush it. Afterwards, remove the steel ball and transfer the liquid to a new EP tube. Add 200 µL chloroform to each EP tube and mix well, centrifuge at 4 °C, 12,000 rpm for 15 min. After centrifugation, transfer the supernatant (about 400 µL) above the white membrane to a new EP tube, add 400 µL of isopropanol and mix well. Then the sample was placed on ice and incubated for 10 min, following this, the samples were centrifuged at 12,000 rpm for 15 min at 4 °C, and the supernatant was removed. Then 1 mL of 75% ethanol was added and the precipitate was resuspended through repeated pipetting actions to ensure complete dispersion, centrifuge (4 °C, 12,000 rmp, 15 min) and remove the supernatant. Then 75% ethanol was added again to repeat the previous step. Dry the precipitate in the EP tube. After the ethanol volatilizes completely, add an appropriate amount of DEPC-treated water according to the amount of precipitate to dissolve the RNA. Purity and concentration of RNA were assessed with SpectraMax i3x (Molecular Devices, San Jose, CA, United States), and the 260/280 ratio was between 1.8 and 2.0.

### 2.9 Quantitative reverse transcription PCR (qRT-PCR)

Following the manufacturer’s protocol, 1 μg of total RNA was reverse-transcribed into cDNA using the Hiscript II qRT SuperMix for qPCR (R223-01, Vazyme, Nanjing, China). Then 2 µL of cDNA was used in fluorescence quantitative PCR in SYBR Green I using ChamQ Universal SYBR qPCR Master Mix (Q711-02, Vazyme, Nanjing, China). A total of 10 µL of sample volume was added to each well, and the loaded plate was membrane sealed and centrifuged (4 °C, 2000 g, 2 min) and put into a LightCycle 480 System (Roche, Basel, Switzerland). The reaction conditions were set as follows: 95 °C for 30 s; 95 °C for 3–10 s, 60 °C for 10–30 s, 40 cycles; 95 °C for 15 s, 60 °C for 60 s, 95 °C for 15 s. Target gene expression was quantified as fold change using the 2^−ΔΔCT^ method with actin normalization. Primer sequence was listed in Table 2.

**TABLE 2:** List of primers used for qRT-PCR analysis.

Gene name	Forward primer	Reverse primer
Cyp7a1	CCA​CAT​CCT​CCT​GGC​ATT​TA	CAA​GAG​CTG​TAG​ACA​CTG​AGA​C
Cyp7b1	CTC​GTG​AAC​CAC​CCT​TGA​TAA	GTG​TCA​CCA​TGT​TGC​CTT​TG
Cyp8b1	TTT​CTG​AGG​GAG​CAA​GGA​ATA​G	GGA​ATA​AGA​GGA​CCC​AGA​AAC​A
Cyp27a1	ATT​AAG​GAG​ACC​CTG​CGC​CT	AGG​CAA​GAC​CGA​ACC​CCA​TA
Abcb4	CAG​CGA​GAA​ACG​GAA​CAG​CA	TCA​GAG​TAT​CGG​AAC​AGT​GTC​A
Hmgcr	CCA​GAA​GCT​TTC​GTC​AGT​AGA​G	CTC​TGC​TTG​TAG​TCT​CTG​CTT​C
Abcg5	AGA​CGT​TGC​GAT​ACA​CAG​CGA​TG	GTG​CCA​CAG​AAC​ACC​AAC​TCT​CC
Abcg8	CTC​GTG​TGG​TTG​GTG​GTC​TTC​TG	GCC​GTA​GCT​GAT​GCC​GAT​GAC​CA
Shp	CTG​GGA​AGA​AAC​AGG​AAC​AAG​A	GGC​TCA​GAA​GTG​CAT​ACA​GAA​TA
Fgf15	CCC​AGT​CTG​TGT​CAG​ATG​AAG	GAG​GAA​GCA​GTT​GGA​GAC​ATA​G
Fxr1	CCC​TAA​TTA​CAC​CTC​CGG​TTA​TG	TCT​CCT​GCC​AAT​GAC​CAA​TC
Fxr2	TTC​CTC​CTG​CTT​CCT​CTG​TA	TTC​ACC​AAA​CTA​CCC​AAC​TCC
Ostα	TGT​TCC​AGG​TGC​TTG​TCA​TCC	CCA​CTG​TTA​GCC​AAG​ATG​GAG​AA
Ostβ	GAT​GCG​GCT​CCT​TGG​AAT​TA	GGA​GGA​ACA​TGC​TTG​TCA​TGA​C
β-actin	GAG​GTA​TCC​TGA​CCC​TGA​AGT​A	CACGCAGCTCATTGTAGA

### 2.10 Bile acid composition detection

The 39 bile acid mixed standards listed in Table 3 were diluted to the appropriate concentration with methanol, which will be used as the working standard solution. Add an accurately weighed amount of mouse ileum into the EP tube, then add 200 µL of methanol at −20 °C, and shake for 60 s, glass beads (100 mg) were added into the EP tube and the samples were ground with a High-throughput Tissue Grinder (TL-48R, Wonbio, Shanghai, China) (55 Hz, 60 s), repeat at least twice, then ultrasound for 30 min at normal temperature, low-temperature centrifuge at 4 °C (12,000 rpm, 10 min). Take 100 µL of the supernatant into a new EP tube, add 200 µL of water, mix with a vortex, take 10 µL of the supernatant into a new EP tube, mix with 490 µL of 30% methanol solution, and finally filter 300 µL through a 0.22 µm membrane into a detection bottle. Then the samples were detected with Liquid Chromatography (ACQUITY, Waters, United States) and Mass Spectrograph (AB5000, AB Sciex, United States). The injection volume was 5 μL, the column temperature was 40 °C, and the mobile phase was A-0.01% formic acid water, B-acetonitrile. Gradient elution conditions were 0–4 min, 25% B; 4–9 min, 25%–30% B; 9–14 min, 30%–36% B; 14–18 min, 36%–38% B; 18–24 min, 38%–50% B; 24–32 min, 50%–75% B; 32–33 min, 75%–90% B; 33–35.5 min, 90%–25% B. The flow rate was 0.25 mL/min. The ion source temperature is 500 °C, the ion source voltage is −4500 V, the collision gas is 6 psi, the air curtain gas is 30 psi, and the atomization gas and auxiliary gas are 50 psi. Multiple reaction monitoring (MRM) was used for scanning.

**TABLE 3:** Bile acid ion pair.

No.	Abbreviation	Parent ion	Product ion	Retention time (min)
1	alloLCA	375.145	375.145	30.3
2	LCA	375.3	375.3	32
3	isoLCA	375.301	375.301	30.5
4	NorDCA	377.3	377.3	24.6
5	7-ketoLCA	389.311	389.311	24
6	12-ketoLCA	389.314	389.314	24.7
7	6-ketoLCA	389.317	389.317	29
8	Beta-UDCA	391.254	391.254	20.1
9	DCA	391.3	391.3	27.5
10	CDCA	391.301	391.301	26.7
11	UDCA	391.134	391.134	26.7
12	HDCA	391.303	391.303	22.1
13	NorCA	393.211	329.1	16.2
14	DHCA	401.2	401.2	13.7
15	7,12-diketoLCA	403.14	403.14	13.2
16	6,7-diketoLCA	403.3	403.3	24.1
17	Alpha-MCA	407.3	407.3	15.9
18	UCA	407.301	407.301	12.7
19	Beta-MCA	407.302	407.302	16.8
20	CA	407.303	407.303	21.1
21	ACA	407.304	407.304	20.7
22	Beta-CA	407.362	407.362	15.1
23	GLCA	432.401	73.9	27.7
24	GHDCA	448.2	74	15.6
25	GCDCA	448.276	73.9	21.9
26	GUDCA	448.277	73.9	15.2
27	GDCA	448.279	73.9	22.7
28	LCA-3S	455.196	96.9	25.9
29	GCA	464.281	73.9	15.2
30	TLCA	482.223	80	24.1
31	THDCA	498.224	79.9	12
32	TUDCA	498.25	80	12
33	TDCA	498.35	79.8	18.7
34	TCDCA	498.357	79.8	17.4
35	TCA	514.332	79.8	12.7
36	T-alpha-MCA	514.337	79.9	7.31
37	THCA	514.343	79.9	10.2
38	T-beta-MCA	514.346	79.9	7.65
39	CDCA-G	567.525	391.1	20.3

### 2.11 Metagenomic sequencing experiment of intestinal flora

Total genomic DNA of the intestinal flora was extracted according to the manufacturer’s protocol (M9636-02, Omega Bio-Tek, Norcross, GA, United States). Nanodrop 2000 (Thermo Fisher Scientific, Waltham, Massachusetts, United States) and tbs-380 (Turner BioSystems, Sunnyvale, California, United States) were used to detect DNA purity and DNA concentration, respectively. The genomic DNA quality was analysed by electrophoresis on 1% agarose gel (5 V/cm, 20 min). DNA was fragmented on the instrument Covaris M220 (Gene Company Limited, Hongkong, China) with a length of approximately 400 BP. Gene library was established according to the manufacturer’s protocol (NOVA-5144, Bioo Scientific, Austin, Texas, United States). Using a NovaSeq Reagent Kits (20,028,312, Illumina, San Diego, California, United States), one end of the library molecule was complementary to the primer base to synthesize new sequences, and then the double strand was changed into a single strand. One template is fixed on the chip, and the other is washed away. The fixed single strand is bent, complementary to the other primer on the chip, and is fixed to form a “bridge”. After that, PCR amplification is carried out to form double-stranded bridges, repeat the cycle to generate DNA clusters, linearize the DNA amplicon into a single strand, add the modified DNA polymerase and dNTPs with four fluorescent labels, synthesize only one base per cycle, scan the surface of the reaction plate with a laser, read the nucleotide species polymerized in the first round of reaction of each template sequence, chemically cut the “fluorescent group” and “termination group”, restore the 3′end viscosity, continue to polymerize the second nucleotide, and finally count the fluorescent signal results collected in each round to know the sequence of template DNA fragments.

### 2.12 Statistical analysis

Statistical analysis was performed using GraphPad Prism 10. All data were shown as mean ± SEM of three independent experiments. Most of the data were calculated in the following way: t-test was employed to analyze differences between the two groups, analysis of variance (ANOVA) statistical tests were conducted to compare multiple datasets or more than two groups. While the correlation between murine BA level and intestinal flora abundance was analyzed using Spearman’s correlation analysis. Statistical significance was defined as p < 0.05.

## 3 Results

### 3.1 Sevelamer significantly attenuated lithogenic diet-induced cholesterol gallstone formation

**FIGURE 1:**
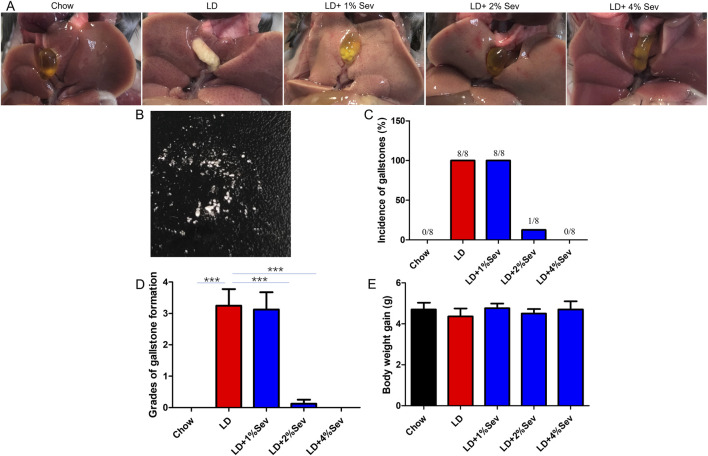
Sev could inhibit the formation of cholesterol gallstones. **(A)** Gallbladder morphology across experimental groups. **(B)** Dried gallstone powder. **(C)** Incidence of gallstones. **(D)** Grades of gallstone formation. **(E)** Body weight gain. n = 8. *p < 0.05, **p < 0.01, ***p < 0.001.

Forty male C57BL6/J mice (6-week-old) were stratified to five experimental groups (n = 8/group): Chow (standard diet), LD (lithogenic diet), and LD with 1%, 2%, and 4% (LD+1%Sev, LD+2%Sev, LD+4%Sev). Following 6-week dietary interventions, the Chow group maintained translucent gallbladder bile without detectable solids (Figure 1A). In contrast, all LD-fed mice developed macroscopic cholesterol gallstones (100% incidence), characterized by viscous bile and gallbladder distension (Figures 1A,C). While LD+1%Sev mice exhibited comparable gallstone prevalence (100%). Notably, the LD+2%Sev group demonstrated marked protection with only 12.5% incidence (1/8 mice), whereas complete prevention was achieved in LD+4%Sev mice (0% incidence) (Figure 1C). Post-desiccation analysis confirmed crystalline deposits in gallstone-positive groups (Figure 1B).

Gallstone scoring (Figure 1D) corroborated these observations: LD and LD+1%Sev groups showed equivalent high scores (p > 0.05), while LD+2%Sev and LD+4%Sev groups exhibited significant reductions compared to LD controls (p < 0.01). There was no significant difference in weight change across all groups throughout the study (Figure 1E), excluding confounding nutritional effects. Based on these findings, the LD+4%Sev regimen (hereafter termed Sev group) was selected for subsequent mechanistic investigations due to its complete inhibition of gallstone pathogenesis.

### 3.2 Sevelamer dramatically lowered bile CSI and total cholesterol

**FIGURE 2:**
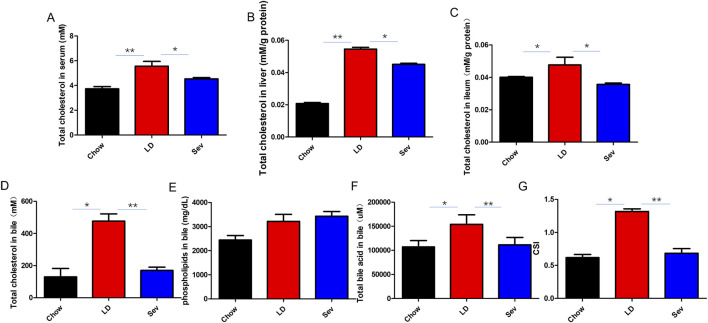
Sev significantly reduced total cholesterol and bile CSI. **(A)** Total serum cholesterol level. **(B)** Total cholesterol level in liver. **(C)** Total cholesterol level in ileum. **(D)** Total bile cholesterol level. **(E)** Bile phospholipid content. **(F)** Total bile acid content in bile. **(G)** Bile CSI. n = 3. *p < 0.05, **p < 0.01, ***p < 0.001.

The development of cholesterol gallstones is closely associated with bile acid and cholesterol homeostasis. The total cholesterol levels in the serum, liver, and ileum of the LD group mice were found to be considerably higher than those of the Chow group, and Sev dramatically decreased the total cholesterol levels of LD group (Figures 2A–C).

The total BAs, cholesterol, and CSI levels in the bile of the LD group were considerably higher than those in the Chow group. The increases in total BAs, cholesterol, and CSI induced by the lithogenic diet were dramatically reversed by Sev intervention (Figures 2D,F,G). There were no appreciable variations in the phospholipid levels in the bile of the three groups (Figure 2E).

### 3.3 Sevelamer ameliorated pathology of gallbladder and liver

**FIGURE 3:**
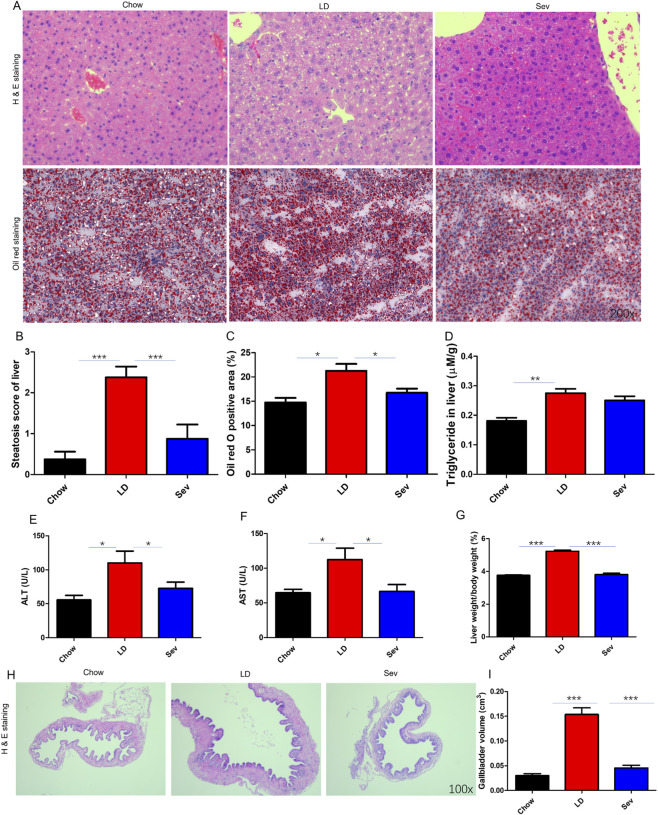
Sev improved liver and gallbladder lesions. **(A)** HE staining and Oil Red O staining of liver tissue sections (x200). **(B)** Liver steatosis score. **(C)** Statistical analysis of Oil Red O staining area in the liver. **(D)** Hepatic triglyceride content. **(E)** Serum ALT levels in mice from each group. **(F)** Serum AST levels in mice from each group. **(G)** The ratio of liver weight to body weight in each group of mice. **(H)** HE staining of gallbladder tissue sections from mice in each group (x100). **(I)** The gallbladder volume in mice. n = 7. *p < 0.05, **p < 0.01, ***p < 0.001.

The LD group exhibited significant lipid vacuoles and cytoplasmic staining compared to the Chow group. The Sev intervention led to the disappearance of white vacuoles, and the staining reverted to normal (Figure 3A). The hepatocyte steatosis scoring system divides the severity of hepatocyte steatosis into three stages. One point is awarded for hepatic steatosis proportions between 6% and 33%, two points for proportions between 33% and 66%, and three points for proportions over 66%. Sev intervention significantly ameliorated hepatic steatosis (Figure 3B). Oil Red O staining showed increased hepatic lipid accumulation and staining area in the LD group compared to Chow group, which was markedly attenuated by Sev treatment (Figure 3C). Hepatic triglyceride levels, determined using a commercial kit, were elevated in the LD group compared to Chow group but were not significantly altered by Sev intervention (Figure 3D). Serum levels of aspartate aminotransferase (AST) and alanine aminotransferase (ALT) were significantly increased in the LD group. Sev intervention effectively normalized these enzyme levels (Figures 3E,F). Mice in the LD group had a significantly higher liver-to-body ratio than those in the Chow group, however Sev intervention significantly decreased liver-to-body ratio (Figure 3G). Gallbladder function and structure were also assessed. The Chow group displayed a thin and transparent gallbladder wall with reduced volume and uniformly distributed muscle layer. In contrast, the LD group showed hypertrophy of the muscular layer and significant thickening of the gallbladder wall. Sev intervention restored normal gallbladder architecture, including muscle layer compaction and wall thickness (Figure 3H). Additionally, the LD group had a considerably larger gallbladder volume than those in the Chow group, which was significantly reduced by Sev treatment (Figure 3I).

### 3.4 Sevelamer increased the expression level of bile acid-related enzymes

**FIGURE 4:**
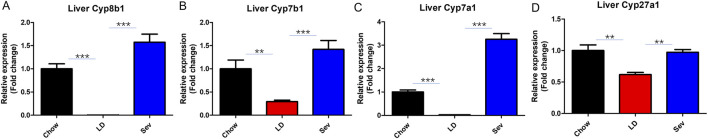
Sev promoted the upregulation of liver bile acid synthesis enzyme expression. **(A)** Relative expression levels of Cyp8b1 mRNA in liver. **(B)** Relative expression levels of Cyp7b1 mRNA in liver. **(C)** Relative expression levels of Cyp7a1 mRNA in liver. **(D)** Relative expression levels of Cyp27a1 mRNA in liver. n = 7. *p < 0.05, **p < 0.01, ***p < 0.001.

The levels of these bile acid synthesis enzymes were significantly lower in the LD group than in the Chow group. Sev intervention could effectively reverse the expression of these enzymes (Figures 4A–D).

### 3.5 Sevelamer prevented the liver from secreting cholesterol

The liver-specific ATP-binding cassette transporters Abcg5/g8 play a critical role in cholesterol secretion into bile canaliculi. To investigate the regulatory impact of Sev on hepatic cholesterol metabolism, we quantified the expression levels of Abcg5 and Abcg8 in livers. Our data revealed that Sev reversed the elevation of Abcg5 and Abcg8 induced by the lithogenic diet (Supplementary Figure S1A,B). These findings implied that Sev may suppress hepatic cholesterol efflux into the bile canaliculus.

Abcb4 (ATP-binding cassette transporter B4) is primarily responsible for hepatic phospholipid secretion. Hmgcr is the primary rate-limiting enzyme for endogenous production of cholesterol. Notably, Sev had no significant effect on Abcb4 expression, whereas the LD group Abcb4 expression was markedly downregulated in comparison to the Chow group. Sev might not affect hepatic endogenous cholesterol production and hepatic phospholipid release (Supplementary Figure S1C). Furthermore, there were no detectable differences in Hmgcr expression among all groups (Supplementary Figure S1D), indicating that Sev neither altered hepatic endogenous cholesterol synthesis nor affected phospholipid secretion pathways.

### 3.6 Sevelamer inhibited intestinal Fxr and downstream gene expression

**FIGURE 5:**
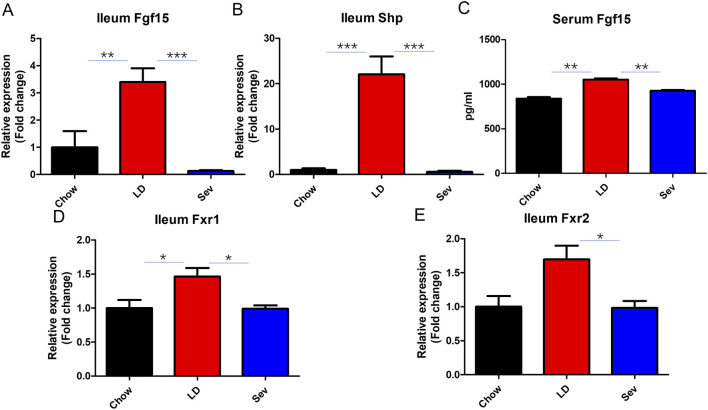
Sev inhibited intestinal Fxr activation and the expression of its downstream genes Fgf15 and Shp. **(A)** Relative expression levels of Fgf15 mRNA in ileum. **(B)** Relative expression levels of Shp mRNA in ileum. **(C)** Serum Fgf15 levels. **(D)** Relative expression levels of Fxr1 mRNA in ileum. **(E)** Relative expression levels of Fxr2 mRNA in ileum. n = 7. *p < 0.05, **p < 0.01, ***p < 0.001.

To investigate the impact of Sev on intestinal Fxr signaling, we analyzed the expression levels of Fgf15 and Shp, key downstream genes of Fxr. The lithogenic diet-induced Fgf15 and Shp expression levels were markedly suppressed by Sev (Figures 5A–C). Similarly, Sev markedly inhibited intestinal Fxr expression (Figures 5D,E).

### 3.7 The inhibitory impact of Sevelamer on gallstone formation could be partially blocked by activating intestinal Fxr

**FIGURE 6:**
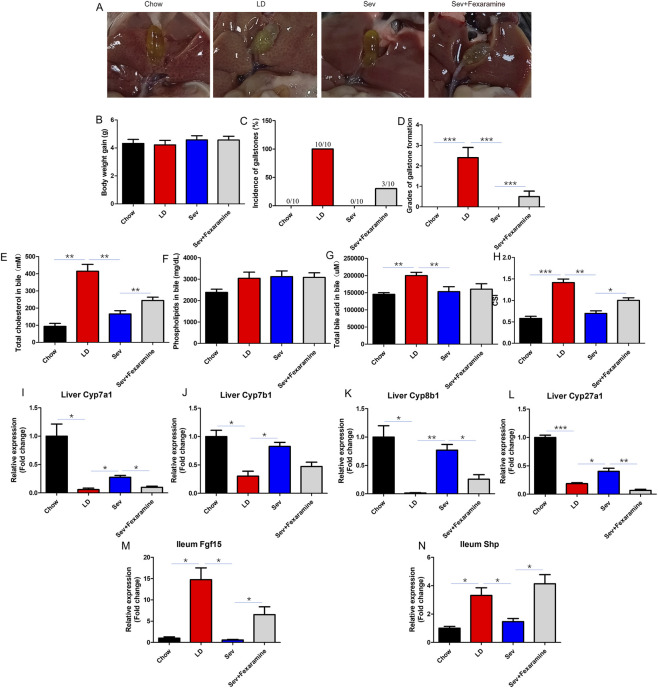
Activation of intestinal Fxr could partially block the effect of Sev on gallstone inhibition. **(A)** Appearance of gallbladders in mice from each group. **(B)** Changes in mouse body weight. **(C)** Gallstone formation rate. **(D)** Gallstone grading. **(E)** Total bile cholesterol content in each group of mice. **(F)** Bile phospholipid content. **(G)** Total bile acid content in bile. **(H)** Calculate the bile CSI of each group. **(I)** The relative expression level of Cyp7a1 mRNA in liver. **(J)** Relative expression level of Cyp7b1 mRNA in liver. **(K)** Relative expression level of Cyp8b1 mRNA in liver. **(L)** Relative expression level of Cyp27a1 mRNA in liver. **(M)** Relative expression level of Fgf15 mRNA in ileum. **(N)** Relative expression level of Shp mRNA in ileum. n = 7. *p < 0.05, **p < 0.01, ***p < 0.001.

According to early experimental findings, Sev could prevent CGs formation by suppressing intestinal Fxr activity and downstream pathways. To further clarify the role of intestinal Fxr in this process, we performed a rescue experiment using an intestinal Fxr agonist (Fexaramine) via intragastric administration. The gallstone formation rate was 0% in the Chow group, 100% in the LD group, 0% in the Sev group, and 30% in the Fxr agonist group. The four groups did not differ in their weight changes (Figures 6A–C). The Fxr agonist can partially counteract Sev’s effect to prevent the formation of CGs (Figure 6D).

The LD grouop bile had significantly higher amounts of total BAs, cholesterol, and CSI than that of the Chow group, which could be considerably decreased with Sev intervention (Figures 6E,G,H). Fxr agonists could increase CSI and total cholesterol levels while partially blocking the effect of Sev on the development of CGs. There was no discernible change in the phospholipid levels across the four groups (Figure 6F).

Cyp8b1, Cyp7b1, Cyp7a1, and Cyp27a1 expression levels were substantially lower in the LD group than in the Chow group. The Fxr agonist might partially inhibit the stimulating impact of Sev on the expression of bile acid enzymes such as Cyp8b1, Cyp7a1, and Cyp27a1 (Figures 6I–L).

When Fgf15 and Shp expression levels in ileum were examined, it was found that the LD group had considerably higher levels of these genes than the Chow group. Sev could suppress their expression, and the Fxr agonist might prevent Sev’s regulatory actions on Shp and Fgf15 (Figures 6M,N).

### 3.8 Sevelamer upregulated bile acid levels inhibiting Fxr activity

**FIGURE 7:**
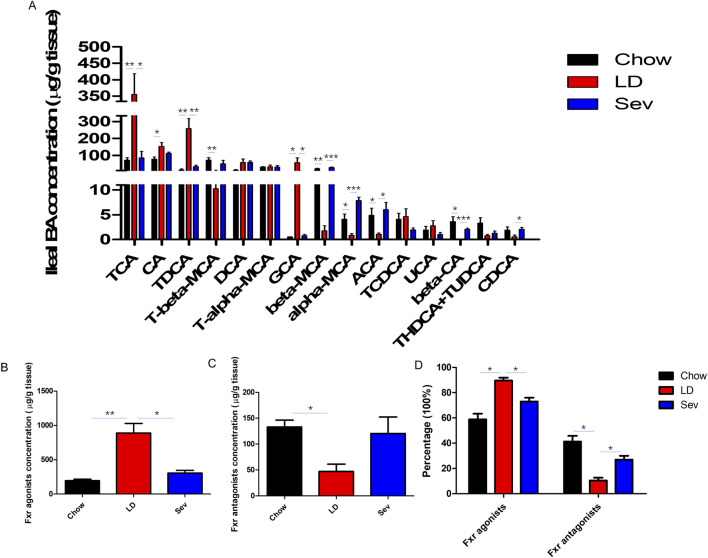
Sev upregulated bile acid levels inhibiting Fxr activity. **(A)** Top 15 level bile acids. **(B)** The absolute concentration of bile acids activatig Fxr in each group of mice. **(C)** The absolute concentration of bile acids with Fxr antagonistic effects in each group of mice. **(D)** The percentage of bile acids in each group of mice that activate or antagonize Fxr. n = 4. *p < 0.05, **p < 0.01, ***p < 0.001.

We analyzed the top 15 level bile acids (Figure 7A). The LD group had significantly higher levels of TCA, CA, TDCA, and GCA than the Chow group, but significantly lower levels of T-beta-MCA, alpha-MCA, beta-MCA, ACA, and beta-CA. Sev might dramatically reduce the levels of TCA, TDCA, and GCA while raising the levels of alpha-MCA, beta-MCA, ACA, beta-CA, and CDCA.

Intestinal BAs were classified as BAs activating Fxr and BAs inhibiting Fxr according to their distinct regulatory effects on Fxr. The LD group had considerably higher concentrations and percentages of BAs activating Fxr than the Chow group, according to calculations of the absolute concentrations or percentages of the two types of BAs in each group. Sev could dramatically lower the percentages and absolute quantities of BAs activating Fxr. Sev could considerably increase the proportion of bile acids inhibiting Fxr (Figures 7B–D).

### 3.9 Multigroup comparison and bile acid correlation analysis were performed at genus level

We selected bacteria datasets linked to cholesterol and BAs and identified species with substantial intergroup differences in order to perform multiple group comparisons at the genus level in the Chow, LD, and Sev groups in order to investigate the relationship between the microbiota and BAs. The species names at each taxonomic level are shown on the vertical axis.

**FIGURE 8:**
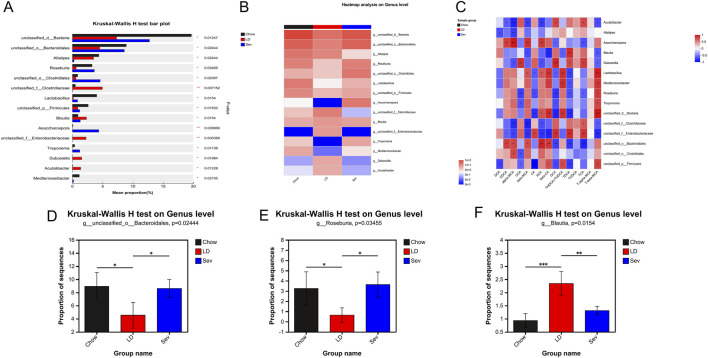
Multigroup comparison and bile acid correlation analysis were performed at genus level. **(A)** Multi-group comparison of species differences in the gut microbiota at the genus level. **(B)** Heatmap of the differences in gut microbiota at the genus level among the groups. **(C)** Correlation analysis between environmental factor bile acids and differential gut microbiota. **(D–F)** Post-hoc analysis of inter-group differences for individual microbiota. n = 4. *p < 0.05, **p < 0.01, ***p < 0.001.

The x-axis shows the abundance of a certain species in the sample, and different colors denote distinct groups. This allows for multiple group comparisons of genus-level species differences (Figure 8A). The genus-level community distribution heatmap, which displays species names on the right and sample names at the bottom, shows the abundance of species by color intensity (Figure 8B). Bile acid environmental parameters are shown by the x-axis, while bacterial species are represented by the y-axis. Calculations were performed to determine the correlation coefficient R value and the associated p value. The R values are shown in various colors, and p < 0.05 is indicated by an asterisk. The range of various R values is shown by the color scale on the right side of the panel (Figure 8C).

The top 15 differentially abundant genera were determined by the Kruskal-Wallis rank-sum test. The post-hoc Tukey-Kramer method was then used to compare these genera pairwise, producing bar graphs of pairwise comparisons for individual species. Bacteroidales and Roseburia had significantly lower relative abundances in the LD group than in the Chow group at the genus level, although Blautia had significantly higher relative abundances. While Sev could drastically reduce the relative abundance of Blautia and could significantly enhance the relative abundances of Bacteroidales, and Roseburia (Figures 8D–F).

### 3.10 Intestinal bile acid reabsorption, cholesterol absorption and efflux might not be connected to Sevelamer

It is possible for intestinal epithelial cells to efficiently reabsorb BAs at the terminal ileum. Ostα/β carries BAs from intestinal cells to the portal venous circulation and ultimately to the liver after the majority of BAs are reabsorbed and moved to the basolateral membrane. Ostα and Ostβ expression levels in the ileum were detected by PCR, and the results showed no variations between the groups, indicating that Sev might not have an impact on intestinal bile acid reabsorption efficiency (Supplementary Figure S2A,B).

Intestinal Abcg5, Abcg8, and Npc1l1 expression levels were assessed in order to analyze intestinal cholesterol efflux and absorption (Supplementary Figure S3A–D). Sev might not have a regulatory influence on intestinal cholesterol efflux and absorption in the gallstone model of mice employed in this investigation, as evidenced by the lack of differences in expression levels across the groups.

### 3.11 Sevelamer improved the composition of intestinal BAs

The alterations in ileal BAs in each group were examined. Principal component analysis (PCA) revealed the ileum’s bile acid makeup. R2X was the verified model’s explanatory power, and its value was equal to the sum of the x- and y-axis values. In the event that R2X exceeded 0.5, the model was considered validated. The R2X in this model was 0.641. There were no glaringly aberrant samples or notable group differences, and all samples fell within the confidence interval (Hotelling T2 ellipse). The closer the bile acid compositions of these samples were to each other, the more clustered the sample distribution points were (Supplementary Figure S4A).

By disregarding the absolute content, the Z-score is a normalized score that is based on the levels of bile acid and is used to compare the relative bile acid content of various groups at the same level. The LD group had higher levels of BAs including GCA, GDCA, NorCA and TDCA than the Chow group, and these levels dramatically dropped after Sev intervention. The LD group had lower levels of BAs including beta-MCA, UDCA and alpha-MCA than the Chow group, but these bile acid levels could be restored following Sev intervention (Supplementary Figure S4B).

The metabolic patterns of bile acids in ileum in each group were identified using hierarchical clustering analysis; the findings were displayed as a data matrix heatmap. To illustrate the variations in the data, a visual color gradient was used. Compared to the Chow group, the levels of BAs such as CA, GDCA, GCA, NorCA, TDCA and TCA were significantly elevated in the LD group, while Sev intervention significantly reduced the levels of GDCA, GCA, NorCA, TDCA and TCA. In contrast to the Chow group, the levels of BAs such as T-beta-MCA, 7-ketoLCA, beta-UDCA, HDCA, UDCA, alpha-MCA, beta-MCA, ACA and beta-CA were significantly lower in the LD group, and Sev intervention could significantly upregulate the levels of BAs such as 7-ketoLCA, beta-UDCA, HDCA, UDCA, alpha-MCA, beta-MCA, ACA and beta-CA. (Supplementary Figure S4C).

### 3.12 Alpha diversity analysis

The following are indicators of communal richness: Chao, Ace and Sobs. Shannon and Simpson are the indexes that show the diversity of the community. Information about species richness and diversity within the community can be gathered through Alpha diversity analysis. Bar charts were created at the phylum, genus and species levels using the Kruskal-Wallis rank-sum test, a multiple comparison test method. The y-axis showed the index values for each group, while the x-axis showed the group names. A significant difference is indicated by p < 0.05. The four indices of Ace, Chao, Shannon and Sobs all demonstrated that the Sev group was significantly higher than the LD group, and there were no differences between the Simpson indices at the phylum level (Supplementary Figure S5A–E). While the Ace, Chao and Sobs indices revealed that the LD group was considerably lower than the Sev group and the Chow group was significantly higher than the LD group, there were no differences between the Shannon and Simpson indices at the genus level (Supplementary Figure S5F–J). The four indices—Ace, Chao, Shannon and Sobs—all demonstrate that the Sev group is substantially higher than the LD group, and there are no differences between the Simpson indices at the species level (Supplementary Figure S5K). This suggests that Sev can considerably increase the Alpha diversity (both richness and variety) of the gut microbiota in mice produced by lithogenic diets at the phylum, genus, and species levels.

## 4 Discussion

The components of 10 gallbladder gallstone samples in human were tested. Among them, cholesterol accounted for more than 90% in 6 gallstones, indicating that the main type was CGs (Supplementary Figure S6). In this study, a CG model in mice was successfully established, and the gallstone prevalence was 100% after feeding 6 weeks with lithogenic diets. We found that Sev suppressed LD-induced CGs in mice. 1% Sev was ineffective, while 2% and 4% doses were significantly effective. The effect of inhibiting CGs is positively correlated with the concentration of Sev, and 4% has the best effect. Sev could improve BA synthesis by modulating Fxr activity, subsequently reducing cholesterol levels, bile CSI and CGs formation. Oil Red O staining and hepatic steatosis scoring indicated significant improvement in lipid deposition by Sev intervention, while triglyceride analysis showed no significant change after Sev intervention, which could be related to the sensitivity of the method or the batch of the reagent kit used.

In addition, Sev modulated the gut microbiota affected by LD consumption. CGs were induced by lithogenic diets in mice, and the balance of lipid metabolism was significantly damaged by high fat and cholesterol levels. As a result, bile CSI increased, cholesterol crystallisation and gallstone formation occurred.

Inhibiting intestinal Fxr activity can suppress the expression of intestinal Fgf15 and Shp, promote hepatic BA synthesis, lower total cholesterol levels, and reduce hepatic cholesterol secretion, thereby exerting an inhibitory effect on the formation of CGs. Furthermore, inhibition of intestinal Fxr activity can reduce liver lipid accumulation and improve liver function. In summary, the results suggest that inhibiting intestinal Fxr activity can prevent the formation of CGs (Huang et al., 2024; Shen et al., 2023). The significant increase in gallbladder volume in the LD group suggests possible gallbladder distention or enlargement due to lithogenic diets, which could contribute to CGs formation. The reduction in volume following Sev intervention indicated a possible therapeutic effect, such as reducing inflammation or improving gallbladder motility, thereby preventing or reducing CGs formation.

Sev significantly improved liver function and liver lipid deposition. The lithogenic diets led to elevated liver enzymes and increased hepatic lipid deposition in mice. However, Sev intervention was able to restore ALT and AST levels and significantly reduce liver lipid accumulation, potentially providing a therapeutic benefit in CGs formation.

Compared to the Chow group, the expression of BA synthase enzymes such as Cyp7a1, Cyp7b1, and Cyp8b1 in the livers of LD mice was significantly downregulated. Sev suppressed Fxr activity in ileum, leading to the downregulation of Fxr downstream target genes Fgf15 and Shp. This resulted in an increase in the expression of BA synthase enzymes such as Cyp7a1, Cyp8b1, and Cyp7b1, promoting the conversion of cholesterol to BA, lowering total cholesterol levels, decreasing bile CSI and inhibiting the occurrence of CGs. Lithogenic diets induced a downregulation of BA synthesis enzymes in the liver, possibly contributing to cholesterol accumulation and CGs formation (Huang et al., 2024; Shen et al., 2023). Sev could improve BA synthesis by modulating Fxr activity, subsequently reducing cholesterol levels, bile CSI and risk CGs formation.

In the LD group, the expression levels of Fgf15 and Shp were significantly higher than those in the Chow group. Sev could inhibit their expression, and an Fxr agonist could block the regulatory effect of Sev on Fgf15 and Shp, and reduce the expression level of liver bile acid synthesis enzymes such as Cyp8b1, Cyp7a1 and Cyp27a1. This partially blocked the downregulation of the bile CSI by Sev and inhibited the formation of CGs.

Sev could regulate gut microbiota composition and intestinal bile acid pool. Patients with cholelithiasis have dysregulated gut microbiota, with significantly reduced diversity and abundance (Song et al., 2022). Cholesterol levels are positively correlated with the abundance of Blautia, and a high-cholesterol diet can increase the content of GCA (Li et al., 2020). Our data showed that the abundance of Blautia significantly increased in the LD group, and Sev intervention could reduce the abundance of Blautia. Analysis of BAs in ileum indicated that GCA levels were increased in the LD group, and Sev could reduce GCA levels. These results were consistent with the literature (Li et al., 2020). High-fat diets could increase levels of CA, GCA, DCA, and TDCA (Huang et al., 2019; Lin et al., 2019). Our experimental results showed that levels of CA, GCA, and TDCA were significantly elevated in the LD group, and Sev significantly reduced GCA and TDCA levels. The mean level of DCA in the LD group were increased, but without statistical significance, partly supporting our experimental results (Huang et al., 2019; Lin et al., 2019). Furthermore, lithogenic diets could significantly reduce the levels of Bacteroidales (Perez-Matute et al., 2015), and our results indicated that levels of Bacteroidales was decreased in the LD group, and Sev could restore these levels. A large-scale clinical study showed that compared with healthy individuals, patients with CGs had a significantly reduced abundance of the intestinal Roseburia (Wu et al., 2013). Lithogenic diets could affect gut microbiota structure, significantly reducing the abundance of Roseburia (Peng et al., 2020). Berberine could significantly reduce cholesterol levels and increase the abundance of Roseburia, with cholesterol levels negatively correlated with Roseburia abundance (Wu et al., 2020). Our data indicated that Sev could restore the decrease in Roseburia abundance caused by the lithogenic diets. According to the literature and our experimental data, Blautia, Bacteroidales, and Roseburia were closely related to gallstones, and Sev could reduce the abundance of Blautia while increasing the abundance of Bacteroidales and Roseburia.

The gut microbiota is an important component of gut microecology and can directly participate in bile acid metabolism. The diversity of BAs is the result of a joint action between host and gut microbiota (Ramirez-Perez et al., 2017). Gut microbiota participates in the formation of secondary BAs through various mechanisms, including bile salt hydrolase (BSH) and 7α-dehydroxylation enzyme activity (Ridlon et al., 2016; Wahlstrom et al., 2017). The first modification step is the deconjugation of glycine or taurine by BSH(Daly et al., 2021). Almost all known major gut microbiota possess BSH(Jones et al., 2008), with many of these bacteria residing in the ileum and colon (Ridlon et al., 2006). The subsequent 7α-dehydroxylation reaction converts almost all CDCA and CA to LCA and DCA (Kriaa et al., 2019). Compared with the LD group, levels of LCA and DCA in the ileum of the Sev group did not increase. Therefore, it is speculated that the effect of Sev may be unrelated to BSH and 7α-dehydroxylation enzyme activity.

Different BAs have various regulatory effects on intestinal Fxr. Known BAs that activate Fxr include: TCA (Chiang and Ferrell, 2018), CA (Parks et al., 1999), TDCA (Zahiri et al., 2011), TLCA (Ginos et al., 2018), DCA (Jiao et al., 2018; Parks et al., 1999), TCDCA (Chiang and Ferrell, 2018), CDCA (Chavez-Talavera et al., 2017; Jiao et al., 2018; Li et al., 2022; Parks et al., 1999), ACA (Vaquero et al., 2013), GCA (Ginos et al., 2018; Yao et al., 2022). BAs that antagonize Fxr include: T-beta-MCA (Li et al., 2013; Liu et al., 2022; Sayin et al., 2013; Sun et al., 2019), T-alpha-MCA (Thibaut and Bindels, 2022), alpha-MCA (Sayin et al., 2013), beta-MCA (Sayin et al., 2013), HDCA (Kuang et al., 2023; Zheng et al., 2021), UDCA (Mueller et al., 2015), TUDCA (Sun et al., 2018), GDCA (Dai et al., 2011). UCA has no significant effect on Fxr-mediated transcription, as the expression of downstream Shp and Cyp7a1 in Fxr does not change (Xu et al., 2002). We analyzed the differences in the levels of the top 15 BAs among different groups. By calculating the levels of BAs that activate or inhibit Fxr, it was found that the levels of Fxr-activating BAs were significantly higher in the LD group compared to the Chow group, and Sev significantly reduced the levels of Fxr-activating BAs in mice fed a lithogenic diet. The levels of Fxr-antagonistic BAs were significantly lower in the LD group compared to the Chow group, and Sev significantly restored the levels of BAs with Fxr-antagonistic action. This indicated that Sev could inhibit the intestinal Fxr pathway by regulating the composition of the bile acid pool in the gut.

According to the literature, the gut microbiota plays a key regulatory role in the composition of the bile acid pool. Therefore, when BAs are considered as environmental factors and their correlation with the gut microbiome is analyzed, it is shown that Sev may exert its inhibitory effect on cholesterol gallstone formation by decreasing the abundance of Blautia and increasing the abundance of Bacteroidales and Roseburia to enhance the levels of BAs that antagonize intestinal Fxr.

The CG model established in this project simulated a human CG model, and one of the main risk factors for gallstone formation was excess cholesterol. Sev, an inhibitor of intestinal Fxr, promotes the conversion of cholesterol into BAs *in vivo*. These results in mice provide a theoretical reference for the prevention and treatment of human CGs.

Our data indicated that Sev could restore the decrease in Roseburia abundance caused by the lithogenic diets. According to the literature and our experimental data, Blautia, Bacteroidales and Roseburia were closely related to CGs, and Sev could reduce the abundance of Blautia while increasing the abundance of Bacteroidales and Roseburia. Sev might inhibit the formation of CGs by reducing the abundance of Blautia and increasing the abundance of Bacteroidales and Roseburia, thereby raising the levels of BAs with intestinal Fxr antagonistic effects and suppressing the intestinal Fxr-Fgf15 pathway.

Based on the results of this study and existing literature reports, the changes in the BAs profile caused by Sev are likely to be indirectly regulated through the modulation of bacterial communities such as Blautia, Bacteroidales, and Roseburia, thereby affecting the BAs profile. The next step should involve performing intestinal transplants with the bacterial communities such as Blautia, Bacteroidales and Roseburia, in order to verify the direct correlation evidence between each bacterial community and the occurrence of CGs. At the same time, through bile acid targeted metabolomics analysis, the causal relationship between each bacterial community and the changes in the intestinal bile acid pool should be clarified.

From the perspective of the mechanism of inhibiting intestinal Fxr, Sev is similar to the existing drug UDCA. However, in terms of biological safety, Sev is not absorbed when taken orally, does not enter the bloodstream, and is 100% excreted from the body in the form of feces (Rosenbaum et al., 1997). Clinical studies have shown that compared with the placebo group, the incidence of adverse reactions does not significantly change and is not related to the dosage. The biological safety of Sev in clinical application may be better.

## 5 Conclusion

Our results support the hypothesis that Sev attenuates CGs formation. These data indicate that Sev inhibited LD-induced Fxr activation and decreased Fgf15 and Shp expression in the ileum, promoted BA synthesis in the liver, and suppressed cholesterol secretion into the gallbladder. In addition, Sev reversed the LD-induced alterations in the gut microbiota.

## Data Availability

The original contributions presented in the study are publicly available. This data can be found here: NCBI SRA, BioProject PRJNA1417970.
