# Supplementary material for: Sevelamer inhibits the formation of cholesterol gallstones by modulating bile acid metabolism
Source: Front Pharmacol. 2026 Feb 10;17:1737631. doi: 10.3389/fphar.2026.1737631 (PMC12929107; doi:10.3389/fphar.2026.1737631)
Supplement: Supplementary file 1 [file Supplementaryfile1.docx]

Supplementary Material

# Supplementary Figures


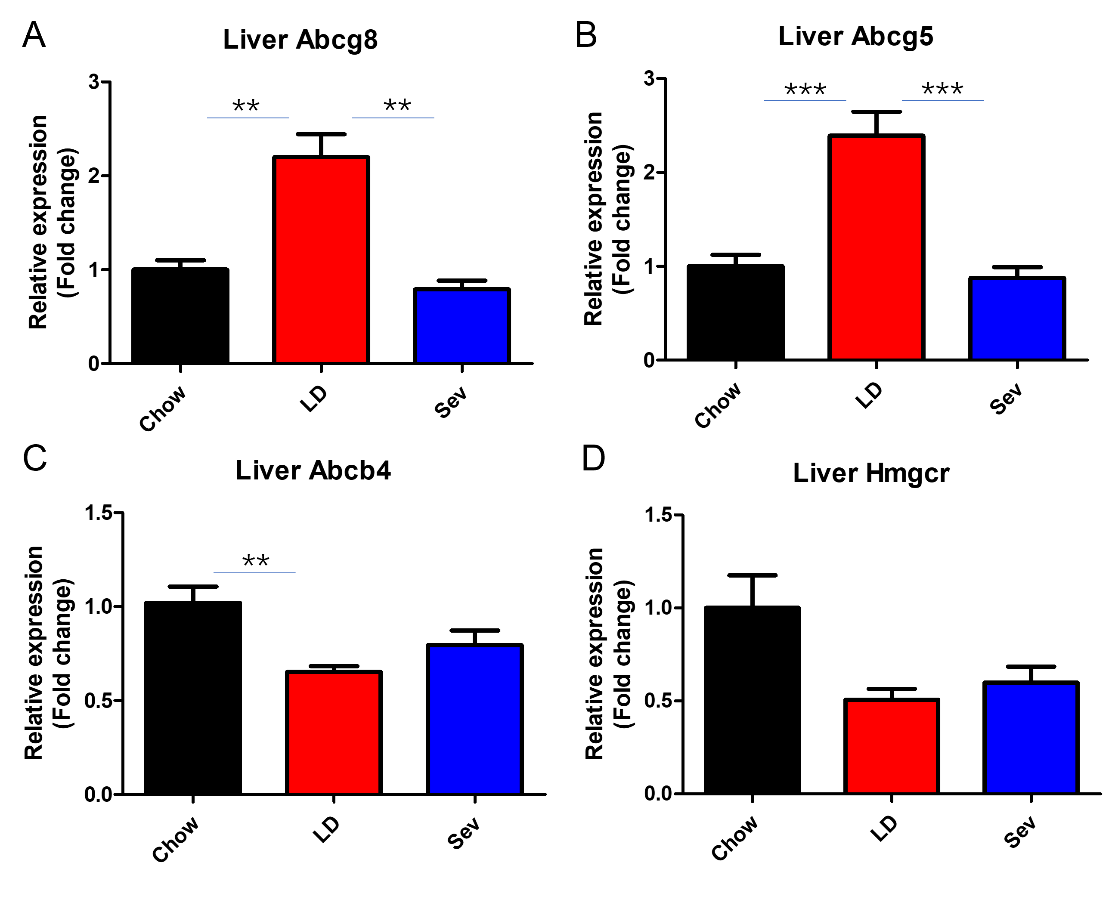


**Supplementary Figure 1. Sev inhibited hepatic cholesterol secretion**A. Relative expression levels of liver Abcg8 mRNA. B. Relative expression levels of liver Abcg5 mRNA. C. Relative expression levels of liver Abcb4 mRNA. D. Relative expression levels of liver Hmgcr mRNA. n=7 *p<0.05, **p<0.01, ***p<0.001.


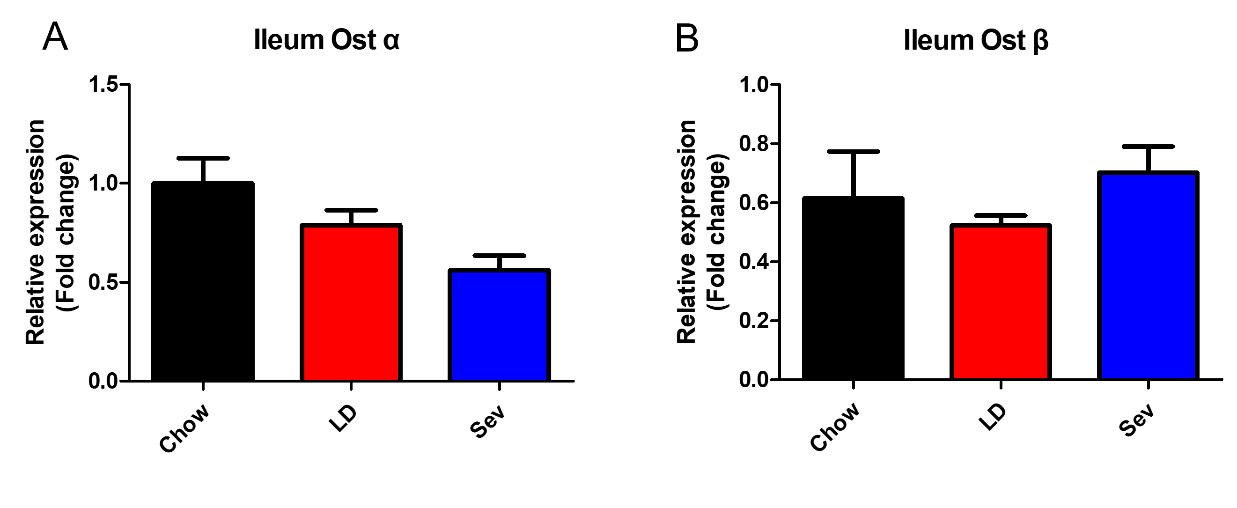
**Supplementary Figure 2. The effect of Sev might not be related to intestinal bile acid reabsorption**

1. Relative expression level of Ostα mRNA in ileum. B. Relative expression level of Ostβ mRNA in ileum. n=7.


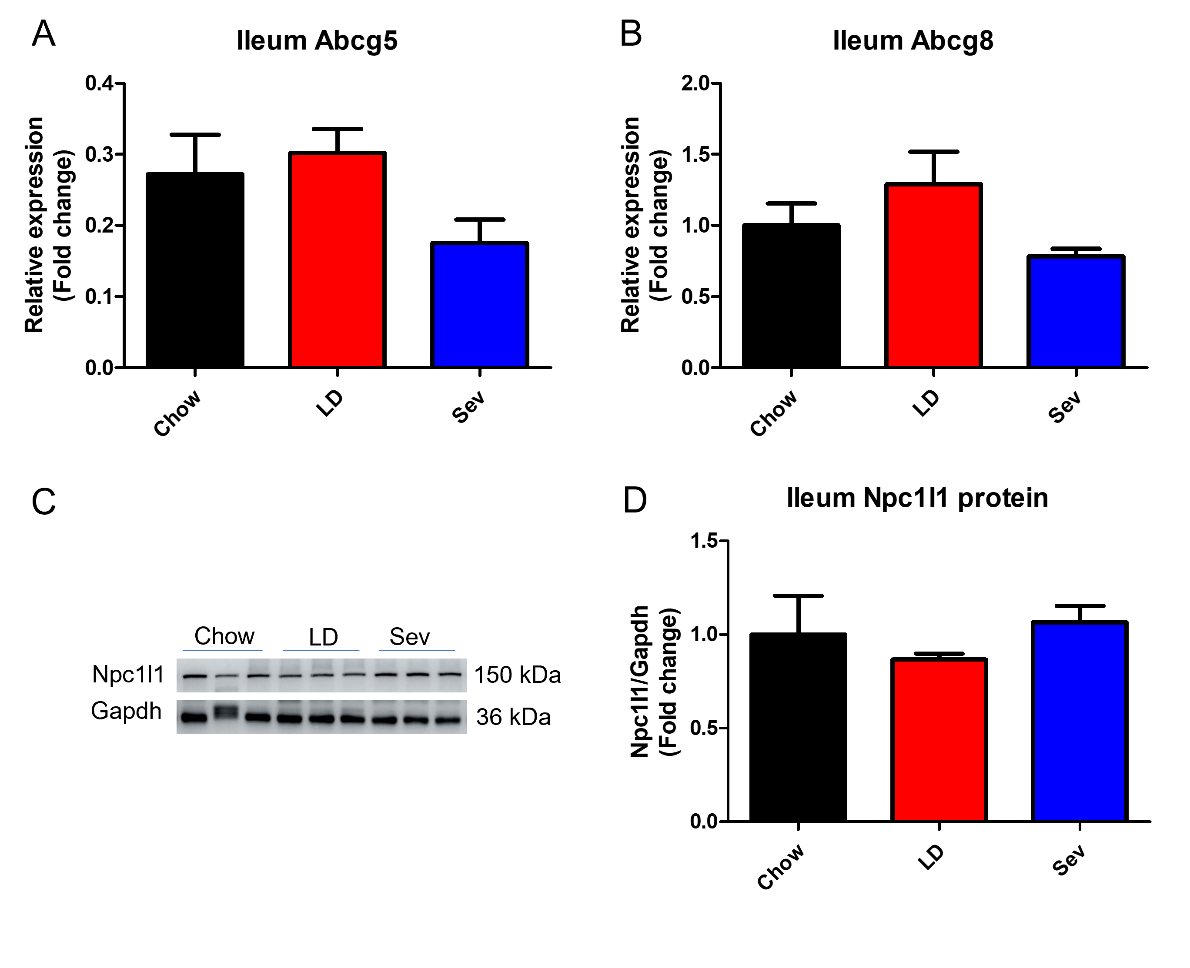


**Supplementary Figure 3. Intestinal cholesterol absorption and efflux might not be connected to Sev**

A. Relative expression level of Abcg5 mRNA in ileum. B. Relative expression level of Abcg8 mRNA in ileum. C. Protein expression level of Npc1l1 in ileum (The Npc1l1 antibody was purchased from Santa Cruz, sc-166802). D. Analysis of relative protein expression level of Npc1l1 in ileum. n=3.

**
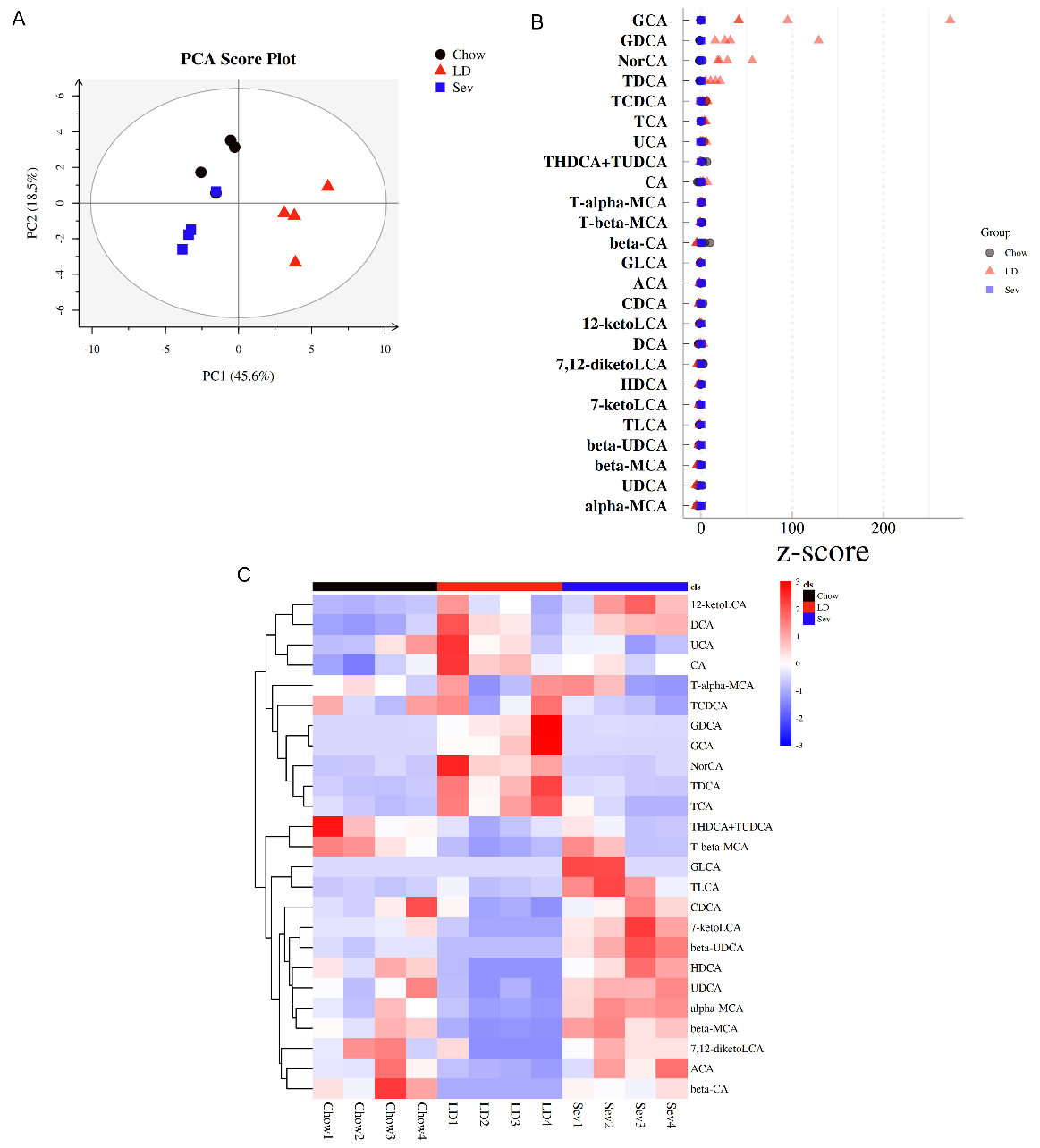
**

**Supplementary Figure 4. Sev improved the composition of intestinal bile acids**

A. Principal Component Analysis (PCA) of bile acid composition in ileum. B. Z-score (standard score) analysis of bile acid levels in ileum. C. Data matrix of bile acid levels in ileum. n=4.

**
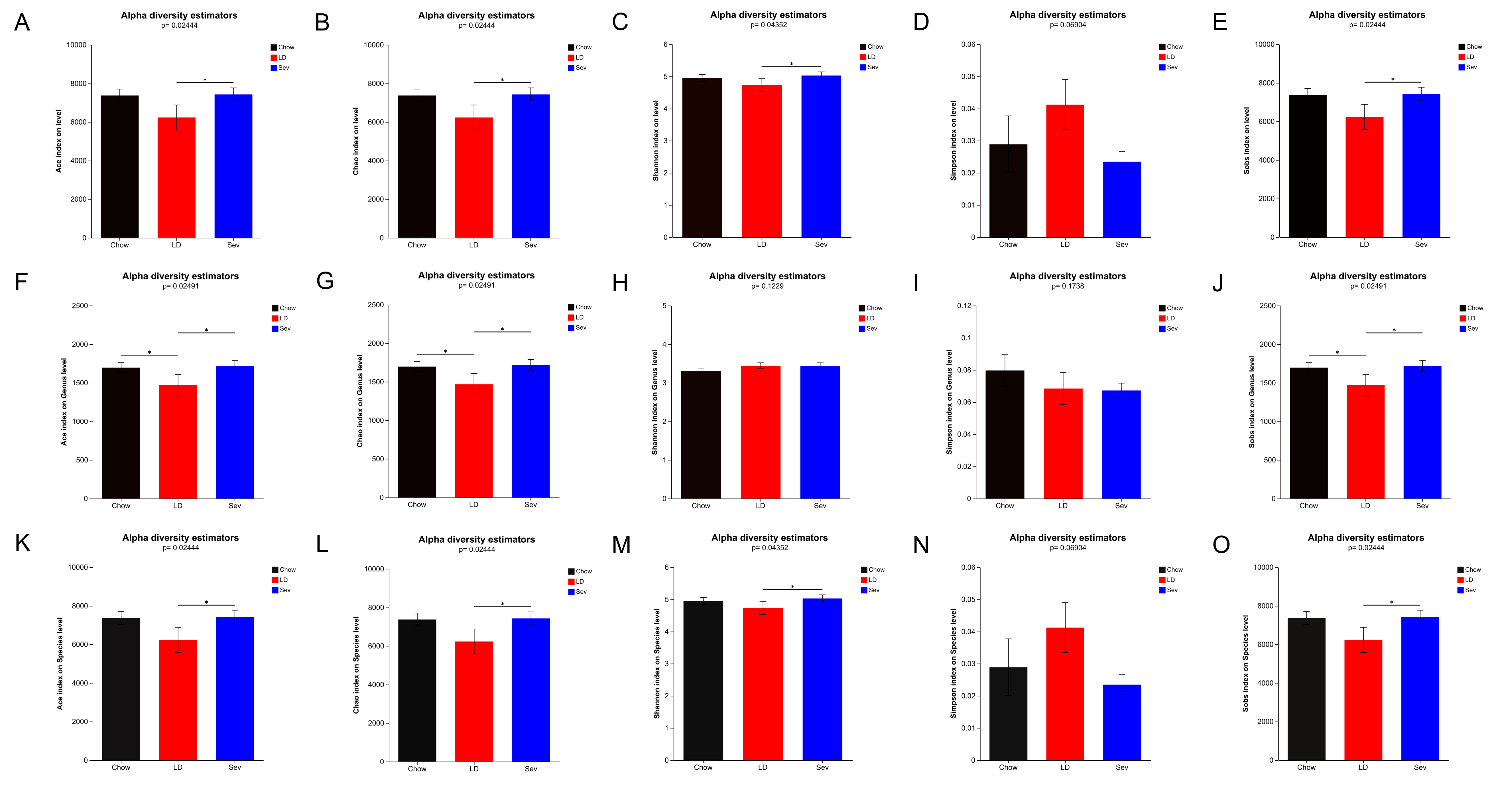
**

**Supplementary Figure 5. Alpha diversity analysis**

A-E. Alpha diversity analysis of the gut microbiota in mice at the phylum level: ace, chao, shannon, simpson and sobs indices. F-J. At the genus level, the Alpha diversity analysis of the gut microbiota in each group of mice was conducted using the ace, chao, shannon, Simpson and sobs indices. K-O. Analysis of Alpha diversity of gut microbiota in mice at the species level among different groups using ace, chao, shannon, simpson and sobs indices. n=4. *p<0.05, **p<0.01, ***p<0.001.

**
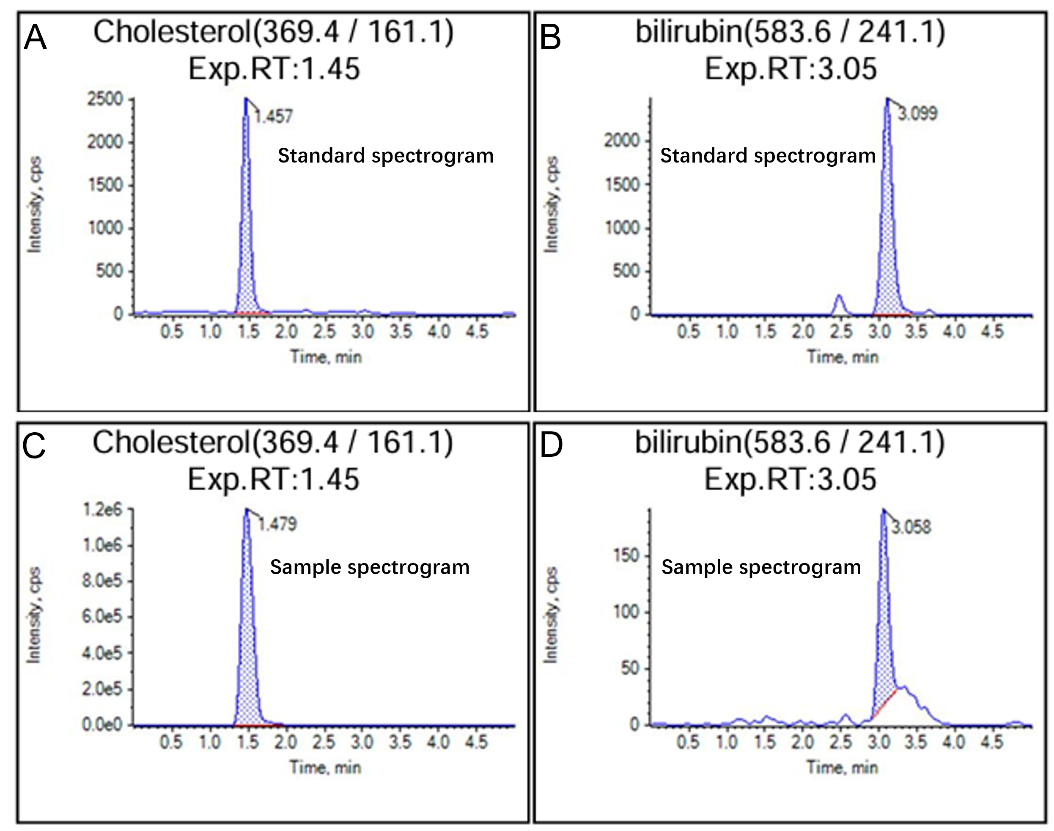
**

**Supplementary Figure 6. HPLC analysis showed that cholesterol was the main component of gallstones**

A. Cholesterol standard spectrogram. B. Bilirubin standard spectrogram. C. Cholesterol sample spectrogram. D. Bilirubin sample spectrogram. n=10.
